# Muscle activation with swinging loads in bench press

**DOI:** 10.1371/journal.pone.0239202

**Published:** 2020-09-17

**Authors:** Atle Hole Saeterbakken, Tom Erik Jorung Solstad, Nicolay Stien, Matthew Peter Shaw, Helene Pedersen, Vidar Andersen

**Affiliations:** Faculty of Education, Arts and Sports, Western Norway University of Applied Sciences, Sogndal, Norway; The University of British Columbia, CANADA

## Abstract

The aim of the study was to compare the EMG amplitude in bench press (stable loads) to bench press using loads moving in anteroposterior and mediolateral directions. Seventeen resistance-trained men, with 9.4±4.7 years of resistance training experience were recruited. After a familiarization session assessing 1 repetition maximum (RM) in the bench press, participants performed: 1) bench press with traditional stable loading 2) bench press with loads (2x5kg) attached as pendulums swinging forward/backwards and 3) left/right in randomized order. The total load was 70% of the 1RM load. Electromyography was measured in the pectoralis major, anterior- and posterior deltoid, biceps brachii, triceps brachii and external obliques. Using stable loads, the pectoralis major demonstrated lower EMG amplitude compared to the two unstable conditions. In the external obliques, the stable conditions demonstrated lower EMG amplitude than the swing in the mediolateral direction, but not the anteroposterior direction. There were no differences between two swinging loads or the three conditions for the triceps brachii, biceps brachii, anterior deltoid or posterior deltoid. In conclusion, swinging in bench press resulted in similar EMG amplitude in the shoulder- and arm muscles, but greater pectoralis and external oblique (only mediolateral swing) activity compared to bench press.

## Introduction

Bench press is one of the most popular and frequently used exercises to gain strength and power in the upper body. In addition to bench press, resistance training enthusiasts often include other chest-press exercises (i.e. dumbbell flies, incline bench press) or use exercise machines in their training routines. Recently, coaches and athletes have included instability within resistance training to strengthen stabilizing muscles and increase stability in the shoulder girdle [1, 2]. Improved joint stability may have advantages when generating force during more stable conditions [3–5].

Several studies have examined the effects of increasing the stability requirements in chest-press exercises [6–8]. One of the most common approaches for providing instability in resistance training is to perform the exercises on unstable surfaces (e.g. Swiss ball, BOSU balls or balance discs). Incorporating high stability requirements (i.e. an unstable surface) decreases the force output, as the body may prioritize stability over force production [6, 7, 9]. However, the existing evidence on EMG amplitude in prime movers and stabilizing muscles lacks agreement, with authors demonstrating increased [1, 10, 11], similar [7, 8, 12] or reduced EMG amplitude [6, 13] depending on the testing procedures and muscles examined.

Practitioners do not typically recommend exercises with major stability requirement in an athletic populations when hypertrophy, absolute strength or power is the primary training goal [14], as combining free-weight exercises standing on unstable surfaces leads to reduced force production [6, 7, 9]. Several coaches have therefore argued that providing a moderate stability requirement, using unstable loads on a stable surface, may be a better approach for strengthening joint stabilizing muscles. Previously, unbalanced water filled loads or loads hanging with elastic bands from the barbell have been used as unstable loads, but without conclusive results [15–18]. Dunnick and colleagues [16] demonstrated similar EMG amplitude examining 60% and 80% of 1RM comparing free-weights bench press with bench press where a portion of the total weights were kettlebells (16 kg) hanging in elastic bands from the barbell. Similar activity in the prime movers was also observed by Ostrowski and colleagues [17] using unstable loads (60% of 1RM) compared to stable loads (75% of 1RM) in bench press. However, greater middle deltoid and biceps brachii activation was observed using unstable loads. By matching the relative intensity (5RM) Lawrence et al. [15] observed greater EMG amplitude in the prime movers during free-weight bench press, compared to kettlebells suspended from the bar with elastic bands, in a sample of powerlifters. Furthermore, biceps brachii activity increased and the 5RM loads were reduced by 32% using the unstable loads. Still, similar EMG amplitude in the shoulder stabilizers (middle- and posterior deltoid, subscapularis, supra- and infraspinatus) between the conditions were observed, which may question whether loads suspended from the bar represent a greater stability requirement or the unique ability of powerlifters to stabilize the shoulder girdle.

The stability requirement of using loads hanging vertically from the barbell was examined in secondary analysis data from Ostrowski and colleagues [17] using unstable loads (60% of 1RM) compared to stable loads (75% of 1RM) [19]. The unstable loads produced two- and three folded bar motion in the anteroposterior and mediolateral direction compared to the stable conditions [19]. Still, the joint stability requirement may not be very large in stable conditions with a resistance trained population [20–22] which may explain the two- and three folded increase in bar motion using unstable loads [19]. Still, Lawrence and colleagues [19] proposed that the unstable loads resulted in a different motor control strategy by better constraining the stabilizing muscles, but the study only included superficial stabilizers and no difference were observed in the deltoids (anterior, middle and posterior), trapezius and latissimus between stable and unstable conditions. However, the prime movers (pectoralis major and triceps brachii) demonstrated lower muscle activation whereas biceps brachii increased EMG amplitude using unstable compared to stable loads [19]. Therefore, using loads swinging, in the anteroposterior and mediolateral directions may increase the shoulder stability requirements. In the previous studies examining unstable loads in bench press, the swinging of the loads were generated by the descending and ascending bar motion [15, 17, 19]. To the authors`best knowledge, no previous studies have examined swinging loads generated by an external force in bench press among resistance trained men. The aim of the study was to compare the EMG amplitude in bench press (stable loads) with bench press using swinging loads (unstable loads) in anteroposterior and mediolateral directions. We hypothesized similar EMG amplitude in the prime movers (pectoralis major, triceps brachii and deltoid anterior), but greater deltoid posterior, biceps brachii and external oblique EMG amplitude using unstable loads.

## Methods

### Design

The study was a cross-over design examining the muscle activation in the upper-body performing bench press with 1) traditional stable loads and two variation combining stable loads with two swinging loads of 5kg each; 2) forward/backwards (anteroposterior) and 3) left/right (mediolateral). Each subject attended two session in the lab. In the familiarization session (session one), 1 repetition maximum (1RM) in bench press was determined. In the same session, the subjects`70% of 1RM was calculated and used to familiarize to the two swinging loads condition (anteroposterior and mediolateral) in a randomized order. The subjects performed five repetitions of the two conditions of swinging loads (5kg swinging on each side) whereas the rest of the load was placed on the barbell as a traditional approach. Three to five days after the familiarization session, the three conditions of bench press were conducted in randomized order in the experimental session with electromyographic (EMG) measurements of the triceps brachii (long head), biceps brachii, anterior deltoid, posterior deltoid, pectoralis major and external oblique.

### Participants

Seventeen resistance-trained men (age 27.3 ± 4.4 years, body mass 81.8 ± 8.0 kg, height 179.8 ± 6.3 cm) with 9.4 ± 4.7 years of resistance training experience were recruited. All subjects were resistance trained with a mean 1RM of 111.3 ± 16.8 kg in barbell bench press. The subjects had to lift their own body weight for one repetition in bench press, perform the exercise with adequate technique (see later), train barbell bench in their weekly training routine in the last six months and be free of injuries to participate. None of the subjects were competing in power-or weightlifting.

### Ethics statement

The study was approved by the Norwegian Center of Research Data (959065), conformed with the University College`s ethical guidelines and the standards of treatment of human subjects in research outlined in the 5th Declaration of Helsinki. All subjects were informed orally, and in writing, of the procedures and gave their written consent to participate before being enrolled in the study. The subjects could withdraw from the study at any time without giving a reason. All subjects were over 18 years (range; 21–33 years).

### Procedures

Before the familiarization session, the subject reported their 1RM in the bench press. From the self-reported 1RM, the warm-up loads (20%, 50%, 70% and 85% of the 1RM) were calculated for the familiarization session. Warm-up sets, which contained 20, 12, 6 and 2 repetitions of the calculated loads (i.e. 20 repetitions with 20% of 1RM etc.), were separated by two to three minutes rest. In the experimental session, the 1RM load achieved in the familiarization session was used to calculate the warm-up loads and the experimental loads.

Four to five minutes after the final warm-up set in the familiarization session, the loads were increased to subjects self-reported 1RM. The loads were then increased or decreased by 1– 5kg until the true 1RM was achieved. The 1RM was achieved within 2–4 attempts with 4–5 minutes separating each 1RM attempt. The subjects started with fully extended elbows and with the head, shoulders and buttocks always in contact with the bench. On command from the test leader, the barbell was lowered and had to touch the chest, without any bouncing, before being elevated until the elbows were fully extended. There was no pause in the lowest position (i.e. powerlifting-style), and the barbell had to be elevated immediately after touching the chest. The foot placement and grip width were self-selected, but the maximal distance between the index finger could not exceed 81cm (powerlifting rules). A 20kg barbell (American Barbell Performance Bearing, Eleiko A/S, Halmstad, Sverige) was used.

**Fig 1:**
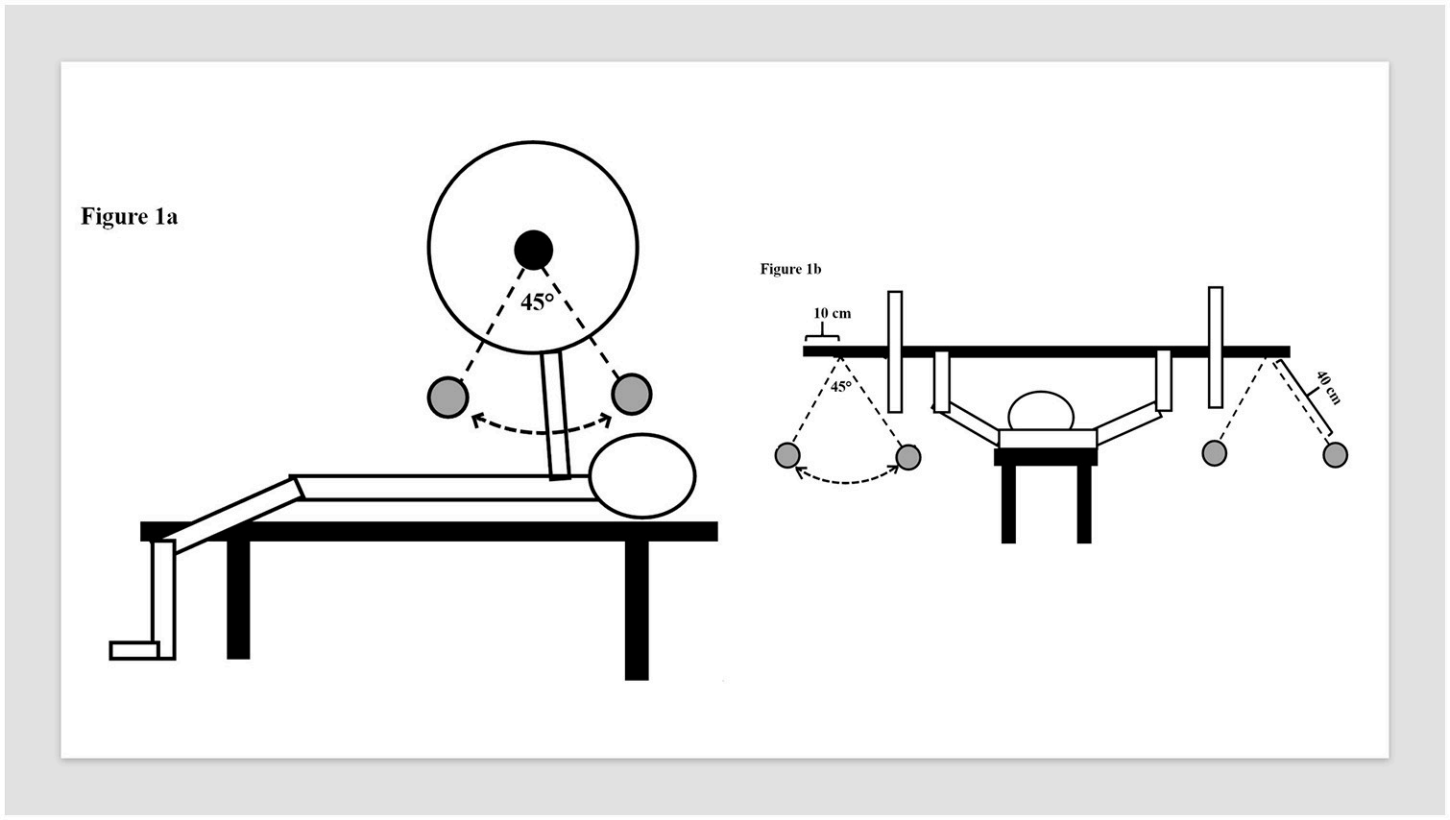
The test-set up for the anteroposterior swing (1a) and mediolateral swing (1b).

Five minutes after the last 1RM attempt, 70% of the 1RM loads were used to familiarize lifting with the swinging loads in the bench press. Importantly, only 10kg (two 5kg weight plates) of the total loads were used as swinging loads. After considerable pilot-testing using different loads, speed and angle of the pendulum, 2 x 5kg was determined as the best option based on the pilot testing. The loads were swinging in the anteroposterior (Fig 1a) in one condition and in the mediolateral plane (Fig 1b) in the second unstable condition, but the procedures were identical. The two 5kg weight plates were attached 10 cm from the end of the barbell using a 40 cm non-elastic rope (Fig 1). The additional load acted as the pendulum and started swinging when subject had the barbell on fully extended elbows. The swinging loads were controlled by two research assistants, and a metronome was used to control the frequency of 90 swings per minute. On the test leader command, two assistants started the pendulum of the loads from the same direction (i.e. superior or left mediolateral). The pendulum swung in total approximately 45° (i.e. 22.5° in each direction from the vertical line) from the perpendicular of the center of the barbell. When the pendulum was correct (i.e. frequency, synchronization and range), the subjects were asked to perform five repetition in a controlled and steady lifting speed without pauses between repetitions. The lifting speed was therefore self-selected to improve the ecological validity. Three to five days after the familiarization session, the experimental session was conducted including measurement of the electromyographic activity. In the experimental session, similar procedures were conducted but without the 1RM testing.

### Measurements

Before placing the surface electrodes (Dri-Stick Silver circular sEMG Electrodes AE-131, NeuroDyne Medical, USA), the skin was shaved, abraded, and washed with alcohol according to previous recommendations [23]. The el-coated self-adhesive electrodes (2-cm center-to-center distance with an 11-mm contact diameter) were placed on the right side of the body as 15 out of 17 participants reported right arm as the preferred throwing arm. The electrodes were placed according to previous studies on the pectoralis major, anterior deltoid, posterior deltoid, biceps brachii, triceps brachii (long head) and external oblique [6, 8, 24, 25]. The EMG was recorded using a Musclelab Data synchronize Unit (Musclelab 6000 system). The EMG signals were sampled at a rate of 1000Hz. To minimize noise from external sources, the raw EMG signal was amplified and filtered using a pre-amplifier (input impedance; 1000GΩ) located close to the sampling point. The common mode of rejection ratio of the preamplifier was 106 dB with a band pass filtered (fourth-order Butterworth filter) with a cut off frequency of 20 Hz and 500 Hz. The EMG signals were rectified, integrated and converted to root mean square (RMS) EMG signals using a hardware circuit network (frequency response 450 kHz, averaging constant 12 ms, total error ± 0.5%). The mean RMS EMG signal of each muscle during the five repetitions was analyzed by Musclelab software (Ergotest Technology AS, Porsgrunn, Norway). Finally, the RMS EMG signals were normalized to the subjects’ five seconds of maximal voluntary isometric contraction (MVIC) according to recommendations [23] and previous studies [26]. Two MVIC trials were conducted for each muscle and separated by 1–2 minutes after the bench press testing. When the subjects were ready, the force was gradually increased until maximal effort and maintained for minimum five seconds. The three seconds with the greatest mean RMS EMG signals were used as the subject`s MVIC. The MVIC exercises were flyes (pectoralis major and anterior deltoid), reverse flyes (posterior deltoid), push-down (triceps brachii), curl (biceps brachii) and sit-ups with rotation (external oblique) [26–28]. The flyes and reverse flyes were conducted seated (90° hip and knee angles) in training machine (Spirit pec fly, Treningspartner, Lierstranda, Norway) with an elbow angle of 170°. The push-down and curl was conducted standing with a 90° angle in the elbows in a cable machine (Own111 cable cross, Sportsmaster, Nesbru, Norway). The subjects used a prone grip and supine grip in the push-down and curl exercises. In the rotated sit-ups, the person lied on the floor with 90° angles in knees and hip. One of the test assistants held the feet while the other held on the shoulders were the manual resistance was applied. The subjects rotated the upper body approximately 45° while generating maximal force. Further details have been described elsewhere [26–28].

To identify the beginning and end of each repetition in the three bench press conditions, a linear encoder (ET-Enc-02, Ergotest Innovation A/S, Porsgrunn, Norway) was used. The time was calculated using a 5-point differential filter with the commercial software v10.4 (Ergotest Innovation A/S, Porsgrunn, Norway). The linear encoder had a resolution of 0.075 mm and counts the pulses with 0.01-second intervals and was synchronized with the EMG measurements using the Musclelab Data synchronize Unit (Ergotest Innovation A/S, Porsgrunn, Norway).

### Statistics

The statistical analyses were conducted using SPSS statistical software (25.0, SPSS Inc., Chicago, IL, USA) and checked for normal distribution using visually inspection of the Q-Q plots and the Shapiro-Wilk test for normality. To examine the differences in EMG amplitude, a repeated analysis of variance (ANOVA) for the three conditions 1) stable loads, 2) unstable loads in the mediolateral plane and 3) unstable loads in the anteroposterior directions was conducted with Bonferroni post-hoc tests. A repeated ANOVA was also used to examine differences in lifting time in the three conditions. The data are presented as mean with the 95% confidence intervals and with Cohen’s *d* effect size (ES) calculated from the mean differences between the conditions divided by the standard deviation of the difference. An ES of < 0.2 was consider trivial, 0.2–0.5 small, 0.5–0.8 medium, and > 0.8 large [29]. The significant level was set to < 0.05.

## Results

In pectoralis major (F = 6.918, p = 0.006), stable loads demonstrated lower EMG amplitude compared to the unstable loads in the anteroposterior direction (p = 0.026, ES = 0.35) and mediolateral plan (p = 0.035, ES = 0.37) with no significant difference between the two unstable loads conditions (p = 1.000). In the external obliques (F = 9.256, p = 0.001), no significant difference was observed between the two unstable conditions (p = 0.429). However, the stable conditions demonstrated lower EMG amplitude than the swing in the mediolateral direction (p = 0.004, ES = 0.66), but not the anteroposterior direction (p = 0.063, ES = 0.48). There were no significant differences between the three conditions for the triceps brachii (F = 3.903, p = 0.053), biceps brachii (F = 0.313, p = 0.669), anterior deltoid (F = 2.731, p = 0.097) or posterior deltoid (F = 2.036, p = 0.157) (Tables 1 and 2). All details are presented in Tables 1 and 2.

**Table 1:** The mean normalized EMG amplitude (% of MVIC) with the 95% confidence intervals.

Muscle	Stable loads	Anteroposterior	Mediolateral	p-values
Pectoralis major	86.54 (72.39–100.69) *	98.08 (80.32–115.83)	98.13 (81.51–114.75)	0.026–1.000
Triceps brachii	40.43 (20.48–60.38)	48.23 (33.42–63.04)	47.60 (30.68–64.52)	0.097–1.000
Biceps brachii	15.88 (7.18–24.59)	17.93 (11.57–24.29)	16.27 (11.23–21.30)	1.000
Anterior deltoid	69.15 (46.22–92.09)	75.21 (51.65–98.76)	74.34 (51.61–97.08)	0.265–1.000
Posterior deltoid	8.70 (4.73–12.69)	10.00 (5.65–14.36)	7.83 (4.63–11.04)	0.079–1.000
External oblique	4.37 (2.54–6.20) #	6.30 (3.67–8.93)	7.37 (4.25–10.49)	0.004–0.429

*significant different than all other conditions (p < 0.05). # Significant different than the mediolateral condition (p < 0.05).

**Table 2:** The mean differences with the 95% confidence intervals of the normalized EMG amplitude comparing stable loads with the swinging loads in the anteroposterior- and mediolateral directions.

Muscle	Anteroposterior	Mediolateral
Pectoralis major	11.54 (1.22–20.85) *	11.59 (0.71–22.48) *
Triceps brachii	7.80 (-2.89–18.49)	7.17 (-1.00–15.35)
Biceps brachii	2.05 (-6.73–10.83)	0.38 (-7.48–8.25)
Anterior deltoid	6.05 (-2.86–14.97)	5.19 (-2.72–13.09)
Posterior deltoid	1.31 (-1.66–4.55)	-2.17 (-4.27–2.54)
External oblique	1.93 (-0.09–3.95)	3.00 (1.02–4.98) *

*significantly different (p < 0.05) compared to stable loads.

The total lifting time was similar between the two unstable conditions (p = 0.127). Using stable loads, the total lifting time was shorter than the anteroposterior condition (p = 0.014), but not compared to the mediolateral condition (p = 0.102).

## Discussion

The main findings of the present study were greater pectoralis major amplitude in bench press during the swinging loads conditions, and similar EMG amplitude in the shoulder- (anterior- and posterior deltoid) and arm muscles (triceps- and biceps brachii). Furthermore, the external obliques demonstrated greater EMG amplitude during the mediolateral swing than during the stable bench press. The novelty of the present study was that instead of using oscillating loads [15–17] suspended from the barbell with elastic bands, the loads were swinging forward/backwards (anteroposterior) or left/right (mediolateral).

Both unstable swinging loads demonstrated ~13% greater pectoralis major amplitude which was not as hypothesized. Greater pectoralis major EMG amplitude was not expected, as increased stability requirement typically reduces or maintains the prime movers activity, while increasing the amplitude of stabilizing muscles [6–8, 16, 17]. However, it is generally accepted that increased stability requirement decreases the force output [6, 7, 9, 30]. Importantly, the pectoralis major`s attachment (from sternum to 6th rib) combined with the multiple functions (flexion, adduction and medial rotation of the shoulder joint) means different parts of the muscle may have a stabilizing role of the glenohumeral joint in the mediolateral and anteroposterior planes, explaining the findings. Furthermore, using the 1RM, with a stable load, to calculate the loads in both stable and unstable loads, may have resulted in greater relative intensity (% of 1RM) in the swinging loads. This may explain the pectoralis amplitude. However, testing 1RM with swinging loads may pose a too large risk of injury for the subjects [13, 31]. Furthermore, the present study used the same procedures as Dunnick et al. [16] (i.e. calculate the loads from the stable 1RM results). However, similar pectoralis major amplitude was observed between stable and unstable loading (16kg Kettlebells hanging from the barbell with elastic bands) using resistance trained subjects as the present study [16].

In contrast to the hypothesis, swinging loads did not increase the biceps brachii amplitude as previous studies have demonstrated [15, 17]. Biceps brachii is one of several stabilizers of the glenohumeral joint [32]. However, Lawrence et al. [15] and Ostrowski et al. [17] used “bouncing” loads (e.g. attaching plates via elastic bands to a barbell) as unstable loads in contrast to the present study using “mechanical” controlled swinging loads. It could be speculated that the procedures, especially the amount of instability in Lawrence et al. [15] and Ostrowski et al. [17] may represent a different form of stability requirements in the biceps brachii. A unstable bouncing load may represent a more unpredictable load than the swinging loads in the present study. Lawrence et al. [15] included several of the glenohumeral joint stabilizers, but with the exception of biceps brachii, no differences in EMG amplitude was observed in them comparing stable and unstable loads. However, the biceps brachii was not investigated. Secondary analysis of the data from the comparison of 60% of 1RM in unstable loads with 75% of 1RM in stable loads [17] demonstrated greater mean biceps brachii amplitude during unstable loads [19]. Greater biceps brachii amplitude could be a strategy to control mediolateral and anteroposterior directions which were supported by the authors analysis of the barbell movement [19]. The unstable loads resulted in greater movement in the mediolateral and anteroposterior directions, but not in the primary direction of the press (vertical direction). Irrespective of this, the unstable loads demonstrated less movement which could indicate that the lifters used more effort in controlling and stabilizing the loads. Further studies should, therefore, examine the effects of different approaches to include instability in resistance training.

In partial agreement with the hypothesis, swinging loads did not increase the EMG amplitude of the posterior deltoids, but similar EMG amplitude in the anterior and posterior deltoids was observed. This is partly in contrast to what previous studies have demonstrated [15–17]. Still, the findings from the deltoid amplitude are not conclusive [15–17]. For example Lawrence et al. [15] demonstrated similar middle and posterior deltoid EMG amplitude, but lower anterior deltoid amplitude using unstable loads compared to stable loads. Furthermore, Ostrowski et al. [17] demonstrated similar anterior deltoid EMG amplitude as the present study, but greater middle deltoid amplitude using unstable loads. Finally, Dunnick et al. [16] demonstrated similar middle deltoid EMG amplitudes. In the present study, all subjects were resistance trained men. Conversely, Lawrence et al. [15] included powerlifters and tested each set to fatigue (5RM). The present study and Dunnick et al. [16] examined a given number of repetitions (3 and 5) of the percentage of 1RM (60–80%) to non-fatigue. Lastly, Ostrowski et al. [17] used different testing intensities (60% and 75% of 1RM using unstable and stable loads). Higher testing intensities (i.e. % of 1RM) have demonstrated increase EMG amplitude [16, 33, 34]. The different testing intensities may, therefore, explain the different findings between the studies.

In triceps brachii, similar activity was observed across conditions, as hypothesized and in line with previous studies [15–17]. Unstable loads, either swinging in the present study or bouncing [15–17], may therefore not affect the triceps brachii EMG amplitude significantly. These findings are partly in according to bench press studies examining unstable surfaces [7, 8]. However, Saeterbakken et al. [6] demonstrated lower triceps brachii amplitude using an unstable surface. It could be argued that unstable loads may be a better approach to include instability in resistance training. Lifting unstable loads on a stable surface, in contrast to stable loads on an unstable surface, has been proposed by several coaches and athletes as more applicable to real-life scenarios as force is very rarely generated being on an unstable surface. However, generating force with unstable loads (i.e. an opponent in soccer) on a stable surface occurs more frequently.

In the external oblique, the EMG amplitude increased with the mediolateral swing compared to the stable conditions, whereas only a statistical tendency (p = 0.063) was observed between stable loads and swing anteroposterior. This weakened our hypotheses and similar findings have been observed during bench press on an unstable surface [6, 10]. As the external oblique stabilize and stiffen the body by increasing the abdominal pressure and preventing rotation of the contralateral side, it was unsurprising that the subjects had to increase the external oblique during the mediolateral swing. Importantly, the distance between the feet was identical across all conditions as the feet are typically used to stabilize the trunk during bench press. Therefore, few other muscles could stabilize the trunk during the mediolateral direction.

The present study had some limitations which need to be addressed. Firstly, the subjects had only one familiarization session. However, all subjects were resistance-trained and had extensive barbell bench press experience. Secondly, the sets were not tested to fatigue and used similar absolute intensity (70% of 1RM in stable bench press). Thirdly, only two 5 kg were used as swinging unstable loads, independent of their individual strength level. Using larger loads may result in greater stability requirements. Lastly, using surface EMG, there is always an inherent risk of crosstalk between the muscles [35]. However, all EMG activity was normalized to the subjects MIVC.

In conclusion, swinging unstable loads in the mediolateral and anteroposterior direction in the bench press resulted in similar EMG amplitude in the shoulder- and arm muscles, but greater pectoralis and external oblique (only mediolateral swing) compared to stable loads in bench press.
